# Characterization of Anti-Ana o 3 Monoclonal Antibodies and Their Application in Comparing Brazilian Cashew Cultivars

**DOI:** 10.3390/antib10040046

**Published:** 2021-11-28

**Authors:** Christopher P. Mattison, Barry Vant-Hull, Ana Cecilia Ribeiro de Castro, Heidi J. Chial, Yvette Bren-Mattison, Peter J. Bechtel, Edy Sousa de Brito

**Affiliations:** 1 Southern Regional Research Center, FPSQ, ARS, U.S. Department of Agriculture, New Orleans, LA 70124, USA; bechtel@gmx.us; 2 Contiguity Consulting, Boulder, CO 80302, USA; barry.vanthull@gmail.com; 3 Embrapa Agroindústria Tropical, Fortaleza 60511-110, CE, Brazil; cecilia.castro@embrapa.br (A.C.R.d.C.); edy.brito@embrapa.br (E.S.d.B.); 4 BioMed Bridge LLC., 3700 Quebec Street, Suite 100-230, Denver, CO 80207, USA; HEIDI.CHIAL@CUANSCHUTZ.EDU; 5 Louisiana State University Health Sciences Center, New Orleans, LA 70112, USA; yve.mattison@gmail.com; 6 New Orleans Louisiana Neuroendocrine Tumor Specialists (NOLANETS), Ochsner Medical Center, Kenner, LA 70065, USA

**Keywords:** cashew nut, allergy, Ana o 3, antibody, ELISA, cross-reactive

## Abstract

Ana o 3 is an immuno-dominant cashew nut allergen. Four monoclonal antibodies to Ana o 3 (2H5, 6B9C1, 19C9A2, and 5B7F8) were characterized by ELISA and in silico modeling. The 2H5 antibody was the only antibody specific for cashew nut extract. In addition to cashew nut extract, the 6B9C1 and 19C9A2 antibodies recognized pistachio extract, and the 5B7F8 recognized pecan extract. All four antibodies recognized both recombinant Ana o 3.0101 and native Ana o 3. ELISA assays following treatment of purified Ana o 3 with a reducing agent indicated that the 6B9C1 and 19C9A2 antibodies likely recognize conformational epitopes, while the 2H5 and 5B7F8 antibodies likely recognize linear epitopes. In silico modeling predicted distinct epitopes for each of the anti-Ana o 3 antibodies. Screening extracts from 11 Brazilian cashew nut cultivars using all four antibodies showed slight differences in Ana o 3 bindings, demonstrating that these antibodies could identify cultivars with varying allergen content.

## 1. Introduction

Food allergies are recognized as an increasingly important public health problem in many parts of the world. Several factors, including culture, genetics, environment, diet, and microbiota, are thought to contribute to the increased incidence of food allergy over the past couple of decades [1]. Food allergies are associated with significant financial, emotional, and social burdens [2,3]. Accurate detection of allergens is essential for the prevention of accidental allergen exposure. Several types of allergen detection tests are available, including nucleic acid amplification, mass-spectrometry, and antibody-based technologies, such as surface plasma resonance biosensors, enzyme-linked immunoassays, and point-of-care devices [4,5,6].

Cashew nuts are produced by cashew nut trees (*Anacardium occidentale* L.) native to Brazil. Cashew trees typically grow to 13 and 65 feet in height for dwarf and wild plants, respectively, and can spread laterally to cover large areas. Other members of the *Anacardiaceae* family include pistachio, mango, poison ivy, poison oak, and sumac trees [7]. Cashew nuts are produced in several parts of the world, including Africa, India, Vietnam, Cambodia, and Indonesia. The fruit of the cashew tree is sometimes called a pseudocarp or cashew apple. When ripe, the cashew apple is edible and produces sweet, tart, and citrus flavors. The cashew nut is kidney bean-shaped and grows from the bottom of the cashew apple. The nut is encased in a shell containing a toxic liquid skin irritant, cashew nut-shell liquid (CNSL), which is rich in cardol, an alkylphenol compound related to a compound found in poison ivy. CNSL is currently under evaluation for several material and therapeutic purposes [8,9]. Cashew nuts undergo several post-harvest processing steps to facilitate shell breaking and to inactivate the CNSL. According to the International Nut and Dried Fruit Council, the United States was by far the world’s largest importer of cashew nuts and imported 153,448 metric tons in 2017.

Cashew nuts are a nutritious and healthy part of a varied diet [10]. Although cashew nuts contain several healthy components, they are also included in a group of eight foods recognized globally as common causes of food allergies. Allergic reactions to cashew nuts are common and often severe [11,12]. Cashew nut allergens have been shown to be relatively resistant to several types of processing [13]. Immunoglobulin E (IgE) from cashew allergic patients can also cross-react with pistachio, mango, and other members of the *Anacardiaceae* family [14]. Cashew nut allergens include the seed storage proteins Ana o 1, Ana o 2, and Ana o 3 [15].

Ana o 3 belongs to the highly conserved 2S albumin family of proteins. A hallmark of the 2S albumins is a conserved framework of cysteine disulfide bonds that help to endow these proteins with a compact structure. The 2S albumins are small water-soluble proteins resistant to physical and enzymatic attack, are thought to function as plant protective proteins, and are commonly identified as immuno-dominant IgE-binding peanut and tree nut allergens [11,13,15]. Similar to other 2S albumins, the Ana o 3 protein is composed of two subunits held together by disulfide bonds and is an immuno-dominant cashew nut allergen [16]. The presence of IgE antibodies to Ana o 3 is a good indicator of cashew nut allergy and have been correlated with severe allergic reactions [17,18,19]. Ana o 3 is a small protein (migrating at approximately 13 kDa under non-reducing conditions) whose solubility properties and resistance to digestion and processing make it a useful target for cashew allergen detection [20]. The 2H5 monoclonal antibody was generated previously using an Ana o 3.0101 peptide (H2N-CQRQFEEQQRFRNCQR−OH) immunogen and has been demonstrated to recognize properly folded and denatured native Ana o 3 purified from cashew extract, improperly folded recombinant Ana o 3.0101 produced in *E. coli*, and Ana o 3 in fermented cashew yogurt [21,22]. More reagents are needed to study immuno-dominant allergens, such as Ana o 3, so we used four distinct immunogens to develop new antibodies towards Ana o 3.0101 and other Ana o 3 isoforms. Here, we characterize four Ana o 3 monoclonal antibodies, including the 2H5 antibody, by comparing their Ana o 3 binding properties and screening a set of Brazilian cashew cultivars for differences in Ana o 3 recognition.

## 2. Materials and Methods

### 2.1. Materials

Bovine serum albumin (BSA) fraction V and flat-bottom clear-well MaxiSorp 96-well plates were purchased from Thermo Fisher Scientific (Waltham, MA, USA). SDS-PAGE precast Novex 4−20% Tris−glycine gels and 4X NuPAGE LDS sample buffer were purchased from Life Technologies (Carlsbad, CA, USA). Pre-stained Precision Plus Dual protein standards were purchased from Bio-Rad (Hercules, CA, USA). Safe Stain protein staining reagent was purchased from Invitrogen (Grand Island, NY, USA). Powdered dry bovine milk (Carnation Brand, Nestle, Arlington, VA, USA) and ‘ready-to-eat’ cashew nuts, other tree nuts, and peanuts were purchased from a local grocery store. Anti-Ana o 3 antibody production and gene sequencing were performed by Genscript (Piscataway, NJ, USA). Secondary infrared dye-labeled secondary antibodies (IRDye 680RD and IRDye 800CW) were purchased from LI−COR (Lincoln, NE, USA). Defatted nut extracts and purified native (from raw and roasted cashew nuts) and recombinant Ana o 3.0101 were generated as described in Mattison et al. (2019) [21]. Monoclonal antibodies against whole cashew extract or against purified native or recombinant Ana o 3 immunogens were generated using Balb/c mice and standard immunization methods by Genscript (Piscataway, NJ, USA). The 5B7F8 clone was chosen because it recognized only Ana o 3, albeit weakly, on a western blot of cashew extract. The other clones were chosen because they had or shared the highest ELISA titer for each of their respective Ana o 3-specific immunogens. Raw cashew nuts for screening Ana o 3 content were obtained from cashew tree cultivars (2017 harvest) grown at the Embrapa Tropical Agroindustry experimental farm located in Pacajus county, Ceará State, Brazil, with the coordinates 4°11′07″ S; 38°30′07″ W and an altitude of 70 m above the sea level. Pacajus county has climate type Aw, according to Köppen’s classification, with an average annual temperature of about 26 °C and average annual rainfall between 1000 and 1500 mm. The predominant soil type is Red Yellow Haplustults using American Soil Taxonomy classification. Cashew trees belong to an in vivo germplasm bank (BAG-Caju Embrapa). The trees were cultivated under the same horticultural practices and the nuts were harvested in the same season. The specific BAG-Caju orchard where the nuts were collected is located in the Experimental Field of Pacajus-CE (04°10′21″ S; 38°27′38″ W) of Embrapa Agroindústria Tropical. The rainfall pattern is characterized by summer/autumn rains. Two well-defined seasons are recognized; one of rains that usually lasts from January to June, with about 90% of the total annual precipitation and characterized by being extremely irregular, and another dry, from July to December, in which the flowering and fruiting of cashew trees occur. The collected raw cashew nuts were dried and shelled, and the adhering testa (husk) was manually removed from the edible cashew nut kernel.

### 2.2. Enzyme-Linked Immunosorbent Assay (ELISA)

Direct ELISA comparing monoclonal antibody binding to four micrograms (4 µg) of purified native Ana o 3, recombinant Ana o 3.0101, or defatted peanut, cashew, pistachio, pecan, walnut, or almond nut extract was carried out using either untreated samples or samples pretreated with DTT (5 mM). Recombinant Ana o 3.0101 was produced in *E. coli* as a fusion to thioredoxin, as described in Mattison et al. (2019) [21]. The recombinant Ana o 3 protein was used in experiments only after the removal of the thioredoxin moiety. Nut extracts were added directly to microplate wells in 50 µL of sodium carbonate buffer (0.015 M Na_2_CO_3_, 0.035 M NaHCO_3_, pH 9.6) and incubated overnight at 4 °C. The next morning, the plate wells were blocked with phosphate-buffered saline (PBST, 10 mM phosphate, 137 mM sodium chloride, pH 7.4, and 0.1% Tween-20) containing 0.1% BSA for one hour at 37 °C. Plate wells were washed three times with PBST, and 50 µL of each monoclonal antibody was diluted to a concentration of 0.35 µg/mL in PBST and added for one hour at 37 °C. Rabbit polyclonal anti-cashew serum was used at a 1:1000 dilution for all experiments. After three washes with 100 µL PBST, a 1:10,000 dilution of donkey anti-mouse IRDye 680-labeled secondary antibody in PBST was added to each well, and the plates were incubated for 1 h at 37 °C. The donkey anti-mouse secondary antibody used in these tests reacts with both the heavy chains and light chains common to most mouse immunoglobulins. Following three washes with 100 µL PBST, the plates were scanned on a LI-COR Odyssey CLX for signal quantification.

Competitive ELISA to purified Ana o 3, purified from ‘ready-to-eat’ store-bought cashew nuts, was performed with plate wells coated with 4 µg of purified native Ana o 3 in 50 µL of sodium carbonate buffer (0.015 M Na_2_CO_3_, 0.035 M NaHCO_3_, pH 9.6) overnight at 4 °C. Following the overnight incubation, the plate wells were blocked with PBST containing 0.1% BSA for one hour at 37 °C. The plate wells were washed three times with 100 µL PBST to remove unbound protein, and 20 µL of PBST was added to each well. During the blocking step, 25 µL of monoclonal antibody diluted to 0.35 µg/mL was pre-incubated for one hour at 37 °C with 25 µL of purified Ana o 3 in a micro-tube at an initial concentration of 1.0 mg/mL or the indicated log-fold dilutions. The 50 µL pre-binding reactions were then added to the plate wells coated with Ana o 3 (containing 20 µL of PBST, as described above) and incubated for one hour at 37 °C. The plate wells were washed three times with 100 µL PBST, and a 1:2000 dilution of donkey anti-mouse IRDye 680-labeled secondary antibody was added. Following a one-hour incubation at 37 °C, the plate wells were washed three times with 100 µL PBST, and the plates were then scanned on a LI-COR Odyssey CLX for signal quantification.

Antibody binding (relative fluorescence units, RFU) plots represent the average of at least four samples and were visualized with ± standard deviation error bars using Microsoft Excel. Statistical significance was determined using analysis of variance (ANOVA) and post-hoc Tukey HSD with alpha/*p*-value of <0.01.

### 2.3. Mathematical Models for Binding

The binding of a fixed concentration of antibody to varying concentrations of immobilized Ana o 3 protein may be described by the Hill, Equation (1) [23]:

(1) $$\left[A:P_{s}\right]=\frac{A_{tot}}{1+{\left(\frac{K_{Ds}}{\left[P_{s}\right]}\right)}^{H}}$$

where [*A:P_s_*] is the concentration equivalent for the surface density of the antibody-protein complex, *A_tot_* is the total concentration of the antibody (free and complexed), [*P_s_*] is the surface concentration of the unbound immobilized protein, *K_Ds_* is the dissociation constant for the surface reaction, and *H* is the Hill coefficient. The Hill coefficient is often taken to indicate the chemical stoichiometry of the reaction equation, so that *H* = 2, for instance, might indicate dimerization of the protein during or prior to the binding. However, a more conservative interpretation is that *H* merely indicates the apparent degree of cooperativity in a complex interaction [24]. When *H* = 1, denoting simple one-to-one kinetics, the Hill Equation reduces to the Langmuir isotherm [25,26]. When the antibody concentration is well below the value of the dissociation constant, the fraction of the complexed protein will be small, and the total protein concentration *P_tot_* may be used in place of [*P_s_*].

In the experiments described here, the solution concentrations of protein used during the immobilization process are used as a proxy for the surface density of the protein. This assumes that the surface density of immobilized protein is proportional to the concentration of protein in the solution during the immobilization step. The *K_Ds_* derived from these experiments is, therefore, not the actual dissociation constant, and care must be taken when deriving other quantities using these values.

The model for the competition experiments assumes equilibrium between the antibody bound to the immobilized protein on the surface and the antibody bound to protein in solution. The surface-based equilibrium is described by Equation (2):

(2) $$K_{Ds}\left[A:P_{s}\right]=\left[A\right]\left[P_{s}\right]$$

where *K_Ds_*, [*A:P_s_*], and [*P_s_*] are designated as in Equation (1), and [*A*] is the concentration of free antibody in solution.

The equilibrium between free antibody, protein in solution (F = fluid), and antibody-competitor complex in solution is described by Equation (3):

(3) $$K_{DF}\left[A:P_{F}\right]=\left[A\right]\left[P_{F}\right]$$

where *K_DF_* is the dissociation constant for the antibody-protein complex in solution, $[A:P_{F}$] is the concentration of the antibody-protein complex in solution, and [$P_{F}$] is the concentration of free protein in solution. The free surface concentration of the immobilized protein is then described by Equation (4):

(4) $$\left[P_{s}\right]=P_{Stot}-\left[A:P_{s}\right]\;≅\;P_{Stot}$$

where *P_Stot_* is the total concentration of immobilized protein (both free and complexed). Assuming that the antibody concentration is significantly less than *K_Ds_* allows for the indicated approximation.

The free concentration of protein in solution is described by Equation (5):

(5) $$\left[P_{F}\right]=P_{F}{}_{tot}-\left[A:P_{F}\right]\;≅\;P_{F}{}_{tot}$$

where *P_Ftot_* is the total concentration of protein in solution (both free and complexed). Assuming that the antibody concentration is significantly less than *K_DF_* allows for the indicated approximation. As the antibody is bound in both surface-protein complexes and solution-protein complexes, its free concentration is described by Equation (6):

(6) $$\left[A\right]=A_{tot}-\left[A:P_{s}\right]-\left[A:P_{F}\right]\;$$

The surface-bound complex [*A:P_s_*] is what is detected via a labeled secondary antibody. Solving Equations (2)–(6) for [*A:P_s_*] yields, Equation (7):

(7) $$\left[A:P_{s}\right]=\frac{A_{tot}P_{Stot}}{K_{Ds}+P_{Stot}+\frac{K_{Ds}}{K_{DF}}P_{Ftot}}$$

It should be noted that both *K_Ds_* and *P_tot_* are in units of solution concentrations as proxies for surface densities. *A_tot_*, *K_DF_*, and *P_Ftot_* are naturally in units of solution concentration.

### 2.4. Methods of Mathematical Analyses

All data was fit to the models using Igor Pro 8.02 (WaveMetrics, Inc., Portland, OR, USA), a data analysis and display software package with extensive capabilities, including curve fitting. Both Equations (1) and (7) may be fit to the Hill Equation [23,24], which is one of the built-in functions in Igor Pro, Equation (8):

(8) $$y=base+\frac{\left(\max-base\right)}{1+{\left(\frac{x_{half}}{x}\right)}^{rate}}$$

In all cases, we have fixed the *base* at zero, and *y* is taken to be the measured RFU signal.

To model Equation (1) using the Hill Equation, the *rate* is either fixed at 1, fixed at 2, or allowed to float. The following quantities were used for the terms in the Hill equation: *x_half_* = *K_Ds_*, *x* = [*P_s_*] = *P_Stot_*, and *max* = *R A_tot_*, where *R* is the conversion between antibody concentration and RFU (generally unknown).

To model Equation (7) using the Hill equation, the *rate* is fixed at −1, and *x* = *P_Ftot_*. The numerator and denominator of Equation (7) must be divided through by (*K_DF_* + *P_Stot_*) to achieve the form of the Hill Equation, so that, Equation (9):

(9) $$\max=\frac{\;R\;A_{tot}P_{Stot}}{K_{Ds}+P_{Stot}}\;$$

and Equation (10)

(10) $$x_{half}=m_{C}=\frac{K_{Ds}+P_{Stot}}{K_{Ds}/K_{DF}}$$

If *K_Ds_* is found by fitting to Equation (1), *m_c_* is found by fitting to Equation (7), and *P_Stot_* is known, then *K_DF_* (the dissociation constant in solution) is found to be:

(11) $$K_{DF}=\frac{K_{DS}\;m_{c}}{K_{DS}+P_{Stot}}$$

### 2.5. Protein and Antibody Modeling

The Ana o 3 model was generated using the pdb search homology modeling application in MOE (MOE 2019.0101, Chemical Computing Group, Montreal, QC, Canada) with the uniprot Ana o 3.0101 sequence (Q8H2B8) [27], a pam250 scoring matrix, and automatic disulfide bond detection. The final Ana o 3 protein model (E value 8.0 × 10^−14^ and EHMMER 7.2 × 10^−13^) lacked eight amino-terminal residues from the predicted Ana o 3 sequence [15] and was generated using the 2LVF pdb structure of the Brazil Nut Ber e 1 2S albumin [28]. Antibody variable fragments were modeled using the Antibody Modeler application in MOE (MOE 2019.0101, Montreal, QC, Canada). For each antibody, the best scoring Fv and CDR template was used to generate model structures, and they were then used to predict interactions with Ana o 3 using the MOE Protein-Protein Dock application.

## 3. Results

### 3.1. Development of Anti-Ana o 3 Monoclonal Antibodies

**Figure 1:**
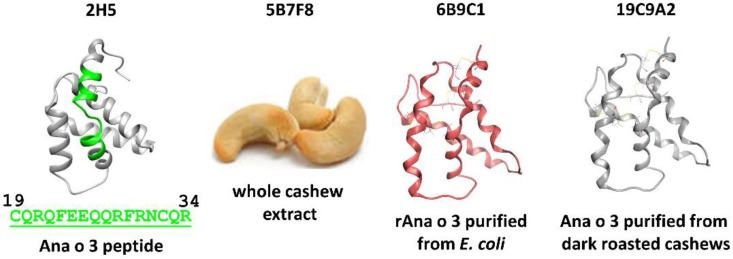
Immunogens used to generate the anti-Ana o 3 monoclonal antibodies included a synthetic Ana o 3.0101 peptide (2H5), whole cashew extract (5B7F8), purified recombinant Ana o 3.0101 produced in *E. coli* (6B9C1), and native Ana o 3 purified from dark roasted cashew nuts (19C9A2).

Several monoclonal antibodies were developed against the Ana o 3 cashew allergen using different immunogens, including whole cashew nut extract, recombinant Ana o 3 (Ana o 3.0101) purified from *E. coli*, and native Ana o 3 purified from dark roasted cashew nuts. After screening mouse sera for high-titer antibodies that recognize Ana o 3, three positive stable clones were chosen for further study. The 5B7F8 clone (isotype IgG1,_K_) was raised against whole cashew nut extract, the 19C9A2 clone (isotype IgG2b,_K_) was raised against Ana o 3 purified from dark roasted cashew nuts, and the 6B9C1 clone (isotype IgG2b,_K_) was raised against recombinant Ana o 3.0101 (Figure 1).

The 2H5 antibody (isotype IgG2b,_K_), which was raised against the Ana o 3 peptide H2N-CQRQFEEQQRFRNCQR−OH and recognizes both the recombinant Ana o 3.0101 and native Ana o 3 proteins, has been described previously [21].

### 3.2. Anti-Ana o 3 Monoclonal Antibody Binding Specificity

**Figure 2:**
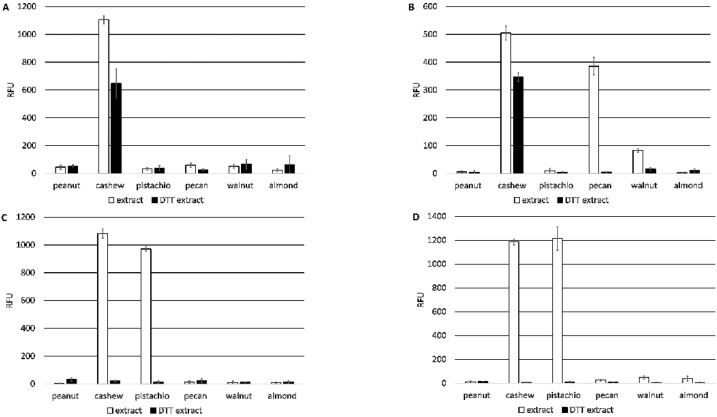
Specificity of anti-Ana o 3 monoclonal antibody binding to native or DTT treated peanut, cashew, pistachio, pecan, walnut, and almond nut extracts. Relative fluorescence units (RFU) are indicated on the *y*-axis. Data represent the average of at least four independent samples, and error bars included show ± standard deviation. Graphs represent ELISA values for (**A**) 2H5, (**B**) 5B7F8, (**C**) 6B9C1, and (**D**) 19C9A2 monoclonal antibodies.

The three new monoclonal antibodies (5B7F8, 6B9C1, and 19C9A2) and the 2H5 antibody were characterized and compared for binding specificity to nut extracts. All four antibodies bound to cashew nut extracts, but only the 2H5 antibody was specific for cashew nut. In addition to cashew nut extract, the 6B9C1 and 19C9A2 antibodies also bound pistachio nut extract at a level comparable to that of cashew nut extract, and the 5B7F8 antibody bound to pecan nut extract and, to a lesser extent, walnut extract (Figure 2). Prior treatment of the extracts with the reducing agent dithiothreitol (DTT) resulted in reduced binding for each of the antibodies. For example, the binding of the 2H5 and 5B7F8 antibodies to cashew extract was lowered, and 5B7F8 binding to pecan extracts was completely disrupted by DTT treatment. Similarly, the binding of the 6B9C1 and 19C9A2 antibodies to both cashew and pistachio nut extracts was completely disrupted by DTT treatment (Figure 2).

**Figure 3:**
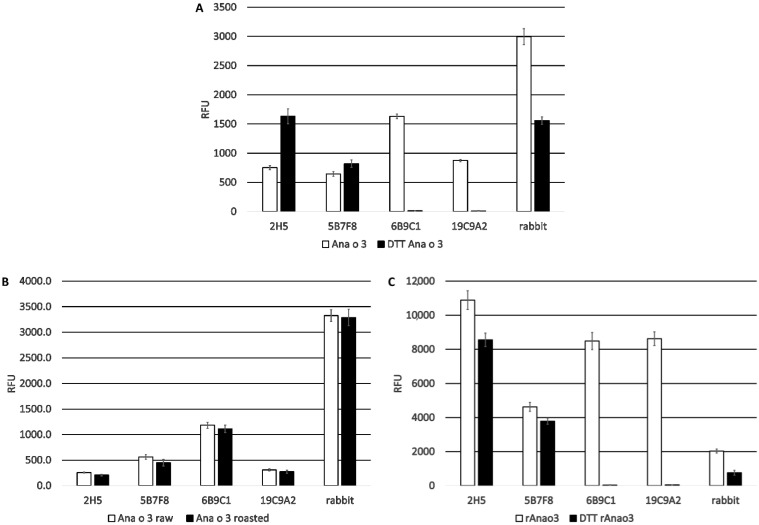
Comparison of monoclonal antibody binding to Ana o 3. Anti-Ana o 3 antibody binding to untreated and DTT-treated purified native Ana o 3 (**A**). Anti-Ana o 3 antibody binding to native Ana o 3 purified from ready-to-eat (RTE) or dark roasted cashew nuts (**B**). Anti-Ana o 3 antibody binding to DTT-treated or untreated purified recombinant Ana o 3.0101 (rAna o 3) (**C**). Relative fluorescence units (RFU) are indicated on the *y*-axis. Data represent the average of at least four independent samples, and error bars included show ± standard deviation.

The four monoclonal antibodies were also evaluated for recognition of purified Ana o 3 (4 µg). Both the 2H5 and 5B7F8 antibodies showed significantly (*p* < 0.01 Tukey HSD) increased recognition of purified native Ana o 3 after DTT treatment (Figure 3A).

For comparison, a rabbit polyclonal serum raised against whole cashew nut extract showed binding to Ana o 3 that was reduced by approximately 50% after DTT treatment. In contrast, the binding of Ana o 3 by the 6B9C1 and 19C9A2 antibodies was completely disrupted after DTT treatment.

Binding to Ana o 3 (4 µg) purified from dark roasted cashew nuts was also compared, but there were no meaningful differences observed among the four monoclonal antibodies or a rabbit polyclonal anti-cashew serum (Figure 3B). All four antibodies recognized recombinant Ana o 3.0101 (4 µg), although relative binding by the 5B7F8 antibody was the lowest (Figure 3C). DTT pretreatment of the recombinant Ana o 3 protein reduced binding by the 2H5 and 5B7F8 antibodies (in contrast to what was observed with the native protein), whereas binding by the 6B9C1 and 19C9A2 antibodies was completely disrupted (Figure 3C). Binding to recombinant Ana o 3.0101 by the rabbit polyclonal serum was relatively weaker and was further reduced following DTT pretreatment, suggesting this polyclonal serum predominantly recognizes conformational epitopes.

### 3.3. Anti-Ana o 3 Monoclonal Antibody Binding Kinetics

**Figure 4:**
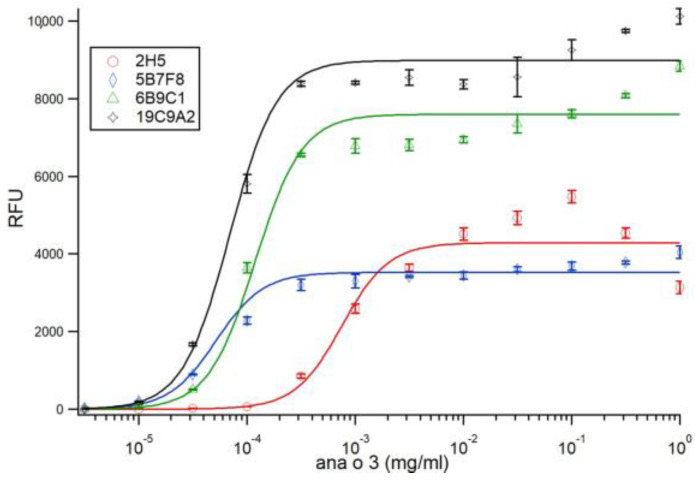
Ana o 3 antibody binding sensitivity characterized by limiting dilution ELISA. Fixed amounts of each antibody were incubated with half-log dilutions of immobilized Ana o 3. Ana o 3 concentration is indicated on the *x*-axis, and relative fluorescence units (RFU) are indicated on the *y*-axis. Data represent the average of at least four independent samples, and error bars included show ± standard deviation. Solid lines represent fits to Equation (1), using a Hill coefficient of 2.0.

The Ana o 3 binding sensitivity among the monoclonal antibodies was characterized by ELISA using limiting dilution. Fixed amounts of each antibody were titrated with half-log dilutions of immobilized Ana o 3. Fitting of the data to Langmuir isotherms (Hill coefficient equal to 1.0) did not yield satisfactory results. Allowing a floating Hill coefficient yielded coefficients between 1.8 and 2.0 for three of the four antibodies and a Hill coefficient of 1.45 for the 5B7F8 antibody. In order to maintain consistency, the data was refit using the Hill coefficient fixed at 2.0 for all antibodies, which resulted in less than a 20% change in fitted *K_DS_* values. Fits for all antibodies with the Hill coefficient fixed at 2.0 are shown in Figure 4, and the calculated *K_DS_* values thus found are shown in Table 1.

**Figure 5:**
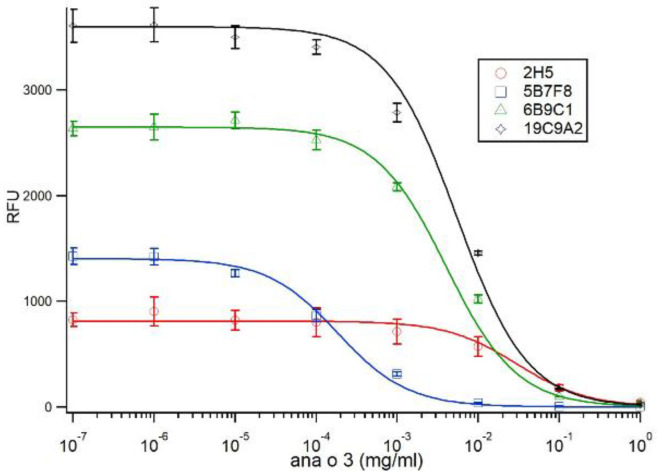
Competitive ELISA comparing antibody binding to purified native Ana o 3 protein. Ana o 3 protein was immobilized to the surface using a concentration of 80 µg/mL. The concentration of competitive Ana o 3 (mg/mL) in solution is indicated on the *x*-axis, and relative fluorescence units (RFU) are indicated on the *y*-axis. Data represent the average of at least four independent samples, and error bars included show ± standard deviation. Solid lines represent fits to Equation (7), via incorporation of Equations (9) and (10).

Competitive binding experiments were performed using fixed amounts of each antibody pre-incubated with log-fold dilutions of Ana o 3 in solution, followed by incubation with fixed amounts of immobilized Ana o 3 (Figure 5).

Fitting of the data to Equation (7) (using the Hill Equation with a Hill coefficient of −1) yielded values for *m_c_* for each antibody, as shown in Table 1. Using the fit values of *K_DS_* for each antibody in Equation (11) allowed the calculation of the solution dissociation constant *K_DF_* for each antibody, as shown in Table 1. The calculated *K_DF_* for each of the antibodies using an estimated molecular weight of 13 kDa for Ana o 3 was 21.7 nM for 2H5, 0.0092 nM for 5B7F8, 0.465 nM for 6B9C1, and 0.365 nM for 19C9A2.

### 3.4. Screening Brazilian Cashew Cultivars for Ana o 3 Content

Antibodies can be useful tools to aid in the detection and characterization of food allergens. The protein profile of 11 cashew cultivars from Brazil was compared by SDS-PAGE, and the four monoclonal antibodies were used to compare Ana o 3 content. The characteristics of each cultivar are listed in Table 2.

**Figure 6:**
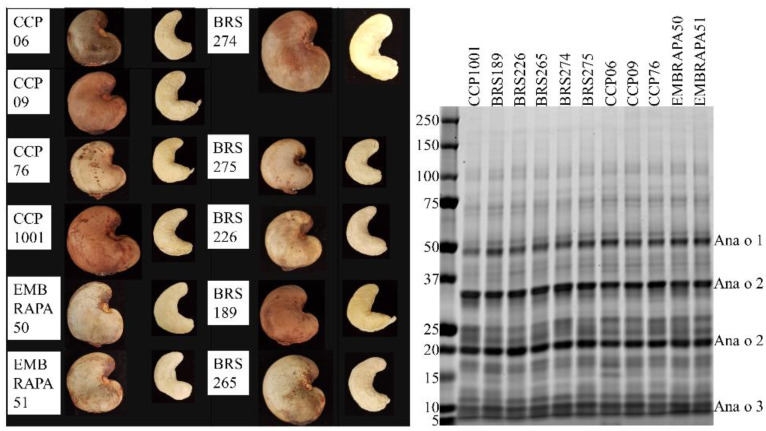
Representative images of unshelled and shelled cashew nuts collected from each cultivar (**left panel**). SDS-PAGE analysis of protein extracts (4 µg) from Brazilian cashew nut cultivars (**right panel**). Molecular weight standards (in kDa) are shown vertically to the left of the gel.

The CCP06, CCP09, CCP76, CCP1001, BRS274, and BRS226 cultivars were collected from nature and cultivated trees. The Embrapa50, Embrapa51, BRS275, BRS189, and BRS265 cultivars are a result of directed crop breeding. Soluble protein from each of the 11 cashew cultivars was evaluated by reducing SDS-PAGE, but the qualitative profile of extracted proteins did not grossly differ among the cultivars (Figure 6).

Each of the cultivars had bands predicted to represent the Ana o 1 protein migrating near the 50 kDa marker, the 35 kDa acidic and 20 kDa basic Ana o 2 subunits, and the approximately 10 kDa Ana o 3 large subunit isoforms. The mature forms of 2S albumins, such as Ana o 3, are generally observed as heterodimers with large and small subunits held together by cysteine disulfide bonds. Treatment of samples with a reducing agent releases the two subunits and allows resolution and visualization of the large carboxy-terminal subunit (7–10 kDa) on SDS-PAGE, but usually not the smaller amino-terminal subunit (3–4 kDa). The 50 kDa band in the CCP1001 cultivar was of lower intensity compared to the same band in each of the other cultivars. There were also differences in band intensities in the lower size range of the gel. Bands in the 25 kDa range varied with the BRS189, BRS226, CCP06, CCP09, and EMBRAPA 51 cultivars having a similar two-band pattern in this region of the gel. The other cultivars contained additional bands leading to a more diffuse banding pattern in this region of the gel. The CCP06 pattern was unique, having a distinct band in the 15 kDa region of the gel that was not present in the other cultivars with the same intensity.

**Figure 7:**
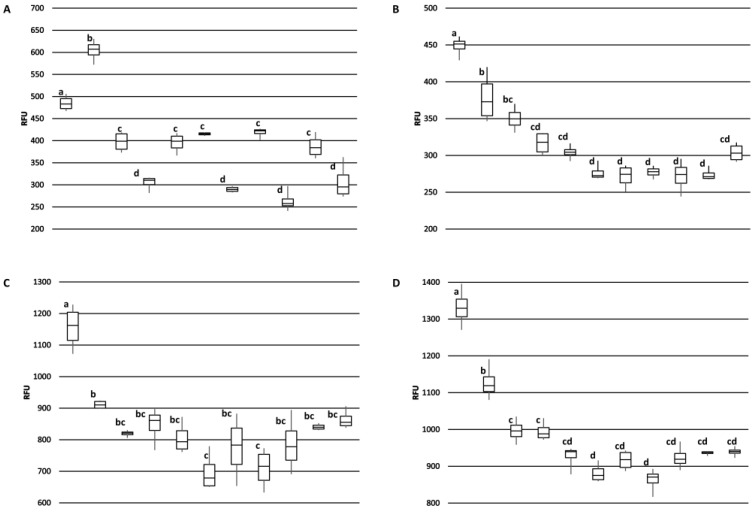
Comparison of Ana o 3 antibody binding among Brazilian cashew nut cultivars. Samples from left to right in each graph are; CCP1001, BRS189, BRS226, BRS265, BRS274, BRS275, CCP06, CCP09, CCP76, RMBRAPA50, and EMBRAPA51. Graphs represent ELISA values for (**A**) 2H5, (**B**) 5B7F8, (**C**) 6B9C1, and (**D**) 19C9A2 monoclonal antibodies. Relative fluorescence units (RFU) are indicated on the *y*-axis. Box plot of antibody binding with the median indicated and error bars included show minimum and maximum values. Statistical significance is indicated overhead each data point by lowercase letters independently for each panel.

The monoclonal antibodies were used to survey for differences in Ana o 3 content among the 11 cultivars by ELISA. Overall, there were no extensive differences in binding among the cultivars, but significant differences in Ana o 3 signal were detected. For example, all four antibodies generated a relatively elevated signal for the CCP1001 cultivar (Figure 7).

One notable exception was that the 2H5 antibody signal was relatively higher for the BRS189 cultivar. The observed Ana o 3 signal was relatively lower for the BRS275, CCP06, and CCP09 cultivars using the 5B7F8, 6B9C1, and 19C9A2 antibodies, while the BRS265, CCP06, and CCP76 cultivars had relatively lower signals when measured with the 2H5 antibody.

### 3.5. In Silico Modeling and Epitope Prediction

**Figure 8:**
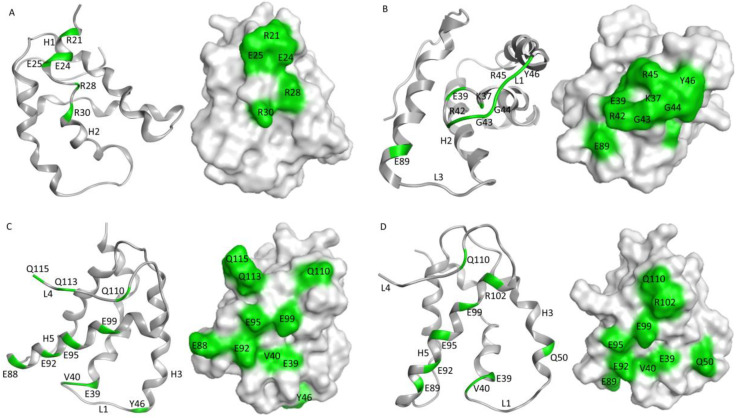
Predicted epitopes for anti-Ana o 3 monoclonal antibodies. Anti-Ana o 3 monoclonal antibody epitopes were predicted using Molecular Operating Environment (CCG) software. Ribbon diagrams and surface exposure models highlighting amino acids in green that are predicted to be important for Ana o 3.0101 recognition by (**A**) 2H5, (**B**) 5B7F8, (**C**) 6B9C1, and (**D**) 19C9A2 antibodies with Ana o 3.0101 loops and helices numbered consecutively.

In silico modeling was used to predict the epitopes of the four anti-Ana o 3 antibodies. Models of each of the antibody variable regions were generated and used to predict interactions with the Ana o 3.0101 protein. The 2H5 antibody was generated with an Ana o 3 peptide corresponding to a sequence within helices I and II of the small subunit of the Ana o 3.0101 protein, and in silico modeling has been used to predict strong interactions between the antibody and the R28 and R34 residues (H2N-CQRQFEEQQRFRNCQR−OH) on the peptide [21]. Consistent with this, the R21, R28, and R30 residues within helices I and II of the small subunit of the Ana o 3.0101 protein were predicted to interact strongly with the 2H5 antibody, along with minor contributions from the R28 and E24 residues within the same helices (Figure 8). The 5B7F8 antibody was predicted to interact with a different region of the Ana o 3.0101 protein. The 5B7F8 antibody interactions were predicted to lie primarily within the loop region following helix 2 in the small subunit with K37, R42, and R45 residues contributing strong interactions to antibody binding (Figure 8).

Epitope predictions for the 6B9C1 and 19C9A2 antibodies were similar to each other. Both the 6B9C1 and 19C9A2 antibodies were predicted to have several interactions with helix 5 residues within the large subunit of the Ana o 3.0101 protein. Both antibodies were predicted to interact with the E92, E95, and E99 residues in helix 5 (Figure 8). Other Ana o 3.0101 residues predicted to interact with both the 6B9C1 and 19C9A2 antibodies include the E39 and V40 residues in the loop between helices 2 and 3.

## 4. Discussion

Four antibodies recognizing Ana o 3 with different affinities and predicted epitope specificities have been developed, characterized, and used to screen several cashew cultivars for differences in Ana o 3 binding. The calculated dissociation constants for Ana o 3 of the antibodies vary in the nano to sub-nano molar range, and application of a specific antibody, or antibody pair, would likely depend upon the desired application they are to be used for. All four of the monoclonal antibodies recognized both improperly folded recombinant Ana o 3.0101 produced in *E. coli* and native properly folded Ana o 3 purified from either ready-to-eat or dark roasted cashew nuts, albeit to different extents. These antibodies are useful reagents that can be utilized in a variety of ways to characterize the immuno-dominant cashew nut allergen Ana o 3. For example, there is a lack of available genetic sequence for cashew cultivars, and in this study, these antibodies were used to screen several cashew cultivars grown in Brazil for differences in Ana o 3 content. Antibody binding of Ana o 3 among the cultivars generally followed the same trend. This is similar to the results obtained by Reitsma et al. (2018) in a thorough comparison of cashew nuts from several different geographic origins [29].

The 2H5 antibody signal was relatively higher for the BRS189 cultivar in particular, whereas binding to this sample by the other antibodies was lower. The 2H5 antibody signal was also relatively higher when compared to the other antibodies for samples from the BRS274, BRS275, CCP09, and EMBRAPA50 cultivars. Whether these observations are a result of genetic differences or alterations due to environmental conditions during growth among the samples tested could, at least partially, be addressed directly through sequencing of the Ana o 3 gene within each cultivar. In addition, a comprehensive multi-year study carefully following growth conditions, as well as biotic and abiotic stresses from multiple cashew cultivars grown in different geographical regions, would help to define what environmental factors affect allergen content. Continued screening of defined cashew cultivars in this manner could lead to the identification of cultivars with reduced allergen content or could lead to breeding strategies enabling reduced allergen content. However, one obvious limitation of this study is that there may be Ana o 3 isoforms with alterations in sequence or conformation that are not detected by these antibodies. Further, it should be noted that without also accounting for the allergenic contribution of the Ana o 1 and Ana o 2 allergens, cashew nuts with reduced Ana o 3 content would still not be safe for those suffering from a cashew nut allergy.

Ana o 3-specific IgE has been documented as a reliable predictor for clinically relevant cashew allergy [17,18,19]. Epitope mapping studies of Ana o 3 peptides have had varied results, and similar to some other food allergens, IgE binding epitopes can be found along the length of much of the protein [15,16]. The monoclonal antibodies described here have predicted epitopes that overlap at some level with published IgE epitopes for Ana o 3. For example, some of the amino acids within the predicted 5B7F8 epitope (K37, R42, and R45) lie within a dominant IgE epitope containing amino acids 35–49 near the carboxy-terminal end of the small Ana o 3 subunit [15,16]. Similarly, strong predicted interactions with amino acids E88/E89 and E92 within the epitopes for the 6B9C1 and 19C9A2 antibodies lie within previously mapped IgE epitopes. Further, weaker 6B9C1 and 19C9A2 antibody interactions with residues E39, V40, and Q110 are also contained within mapped IgE epitopes. One limitation of the epitope predictions described in this research is that only the Ana o 3.0101 protein sequence was used for docking. It is possible that antibodies raised against sources of native Ana o 3, such as the 5B7F8 and the 19C9A2 clones, have reduced affinities towards the Ana o 3.0101 isoform and might thus have been raised against a different Ana o 3 isoform. Future competitive ELISA testing could determine whether these antibodies do, in fact, have epitopes that overlap with and could compete for binding with IgE from cashew allergic sera.

The 6B9C1 clone was derived from the purified recombinant Ana o 3.0101 protein, whereas the 19C9A2 clone was generated using Ana o 3 purified from dark roasted cashew nuts. Both of these antibodies cross-reacted with a protein in pistachio nut extracts. It is possible that the cross-reactivity is due to recognition of the pistachio 2S albumin, Pis v 1, but this was not confirmed in this study. The data presented here suggest the 6B9C1 and 19C9A2 clones recognize a similar conformational epitope, similar to the results obtained here using the rabbit polyclonal anti-cashew sera due to the binding sensitivity after treatment with DTT and in silico modeling results. While the 6B9C1 and 19C9A2 antibodies had a similar predicted epitope and Ana o 3 binding pattern among the assays evaluated here, they were generated with different immunogens. This observation suggests that this region of the protein may be particularly immunogenic and that the structures of the recombinant protein and the native protein purified from heated nuts were likely preserved, at least in this region of the protein. IgE molecules often recognize conformational epitopes, and the 6B9C1 and 19C9A2 antibodies may be used as proxies for IgE binding in future studies. Indeed, this type of approach has been used to study antibody binding to the Ana o 2 cashew allergen [30,31].

In contrast, the 2H5 and 5B7F8 antibodies appear to recognize primarily linear epitopes, based on their enhanced binding when purified native Ana o 3 was treated with a reducing agent. The disruption of the Ana o 3 protein structure with a reducing agent may allow for increased access to the linear epitopes predicted to be recognized by the 2H5 and 5B7F8 antibodies and explain the increased binding. This is not surprising for the 2H5 antibody since it was generated using a peptide, and it binds specifically to cashew nut extract [21]. While the 2H5 antibody was specific for cashew, it may not be specific for Ana o 3. The binding of the 2H5 to purified native Ana o 3 was increased following treatment with the reducing agent, but the treatment of extracts with the reducing agent decreased the 2H5 signal. The 5B7F8 antibody had a similar redox-sensitive binding profile. The binding of 5B7F8 to purified native Ana o 3 was increased by treatment with the reducing agent, but the treatment of cashew extract with the reducing agent decreased the 5B7F8 signal. This suggests that there are 2H5 and 5B7F8 binding contributions from other redox-sensitive components within the extracts. The 5B7F8 antibody was generated using whole cashew nut extract as the immunogen and had some level of cross-reactivity with pecan nut extract and, to a lesser extent, with walnut extract. The linear peptide immunogen used to generate the 2H5 antibody was selected to provide a more specific Ana o 3 antibody, and this is supported by its lack of cross-reactivity with the other nut extracts tested.

The epitope predictions presented here included residues that may impart less important or weaker contact energies, and this may artificially increase the appearance of the predicted allergen-antibody interaction surface. Consistent with the predictions, the 2H5 heavy chain CDR3 contains fewer amino acids than the heavy chain CDR3s for the other antibodies. Similarly, the heavy chain CDR2 of both the 2H5 and 5B8F8 antibodies is shorter than the 6B9C7 and 19C9A2 antibodies. However, the epitope predictions presented here will need to be confirmed using site-directed mutagenesis of recombinant Ana o 3 generated in bacteria or yeast and careful binding studies combining those mutants with each monoclonal antibody. Further, competitive binding experiments with cashew-allergic volunteer sera could be used to confirm whether these antibodies have epitopes that overlap with clinically relevant IgE.

Heating can alter antibody binding to food allergens, depending upon the temperature, duration, and type of heating. High-pressure moist thermal processing has been reported to produce a higher reduction in IgE-binding to tree nut allergens, including cashew nut, compared to dry heat alone [32]. Further, dry heat-induced modifications to the Ana o 3 protein have been identified and characterized [33]. These and other modifications could affect antibody binding to Ana o 3. The antibodies generated here were used to compare binding to recombinant Ana o 3 produced in *E. coli* and native Ana o 3 purified from either ready-to-eat or dark roasted cashew nuts to determine whether there were differences in binding. The 6B9C1 and 19C9A2 antibody clones might be expected to be sensitive to previously described heat-induced modifications on the R91 residue since their predicted epitopes overlap with this region of the protein. However, no obvious difference in binding to the two sources of purified Ana o 3 were observed for any of the antibodies.

In summary, the antibodies described here have varying affinities, recognize at least three different predicted epitopic regions of the Ana o 3.0101 protein that at least partially overlap with established IgE epitopes, and could be used as proxies for IgE binding. Continued use and characterization of the antibodies described here may help foster an improved understanding of the biophysical and structural characteristics of the Ana o 3 protein and may enable the development of improved diagnostic assays for cashew nuts.

## Figures and Tables

**Table 1:** Anti-Ana o 3 Monoclonal Antibody Binding Kinetics.

Antibody	*K_DS_* (ng/mL)	*m_c_* (µg/mL)	*P_Stot_* (µg/mL)	*K_DF_* (ng/mL)
2H5	755 ± 21	30.1 ± 6.4	80 ± 4	282 ± 60.2
5B7F8	52.0 ± 0.5	0.184 ± 0.067	80 ± 4	0.119 ± 0.044
6B9C1	119 ± 2	4.06 ± 0.11	80 ± 4	6.05 ± 0.21
19C9A2	69.6 ± 1.0	5.47 ± 0.11	80 ± 4	4.75 ± 0.14

**Table 2:** Cashew Cultivar Characteristics.

Cashew	Type	Apple Color	Weight	Nut Weight	Kernel Weight	Origin	Parental Relation	Use
CCP 06	Dwarf	Yellow	76.5 g	6.4 g	1.6 g	Collected in Maranguape-CE	CP 06 (Dwarf) Individual phenotypic selection	Rootstock
CCP 09	Dwarf	Orange	87 g	7.7 g	2.1 g	Collected in Maranguape-CE	CP 09 (Dwarf) Individual phenotypic selection	Nut and peduncle for industry
CCP 76	Dwarf	Orange	135 g	8.60 g	1.80 g	Collected in Maranguape-CE	CP 76 (Dwarf) Individual phenotypic selection	Nut and peduncle for table
CCP 1001	Dwarf	Orange	84.6 g	7.0 g	1.90 g	Collected in Maranguape-CE	CP 1001 (Dwarf) Individual phenotypic selection	Nut
Embrapa 50	Semi-Dwarf	Yellow	111 g	11.2 g	2.9 g	CCP 06 (Maranguape- CE) × CP 07 (Pacajus-CE)	Individual phenotypic selection in CP 06 (Dwarf) × CP 07 (common) progeny	Nut
Embrapa 51	Dwarf	Reddish	104 g	10.4 g	2.6 g	CCP 06, CCP 1001, CCP 76, CCP 09, CP 75, CP 10, CP 27 (Maranguape-CE)	Phenotypic selection in a policross progeny	Nut
BRS 274	Standart	Orange	128.6 g	16 g	16.00 g	Beberibe-CE	Individual phenotypic selection in a segregated population	Nut and peduncle for fruit juice industry
BRS 275	Standart	Orange	108 g	11.0 g	11.4 g	CCP 1001 (Maranguape-CE) e CP12 (Pacajus-CE)	Individual phenotypic selection within CCP 1001 (Dwarf) × CP 12 (Giant common) control-pollinated progeny	Nut and peduncle for industry
BRS 226	Dwarf	Orange	102.6 g	9.75 g	2.72 g	Pio IX-PI	Individual phenotypic selection	Nut
BRS 189	Dwarf	Reddish	155.4 g	7.9 g	2.1 g	CCP 1001 (Maranguape-CE) × CCP 76 (Maranguape-CE)	Individual phenotypic selection within CCP 1001 (Dwarf) × CCP 76 (Dwarf) control-pollinated progeny	Peduncle for table
BRS 265	Dwarf	Reddish	118.2	12.5 g	2.6 g	CCP 76 (Maranguape-CE)	Individual phenotypic selection within CCP 76 (Dwarf) open pollinated progeny	Nut and peduncle for industry
